# Improving Lives through Evidence-Based Health Promotion Programs: A National Priority

**DOI:** 10.3389/fpubh.2014.00255

**Published:** 2015-04-27

**Authors:** Richard Birkel, Emily Dessem, Simona Eldridge, Kristie Kulinski, Sue Lachenmayr, Michelle Spafford, Binod Suwal, Albert Terrillion, Mary Walsh, Wendy Zenker

**Affiliations:** ^1^National Council on Aging, Washington, DC, USA

**Keywords:** evidence-based programs, healthy aging, self-management, program sustainability, older adults

The National Council on Aging (NCOA) has set forth on an ambitious course to improve the health and economic security of 10 million older adults by 2020. Specific to older adult health, the promotion of proven, cost effective programs is vital to our collective success. Through our Center for Healthy Aging (CHA), we collaborate with federal, state, and community partners to further the impact and sustainability of evidence-based health promotion programs. Our passion for this work, coupled with our reputation as a valued resource for organizations offering these programs, has afforded us the privilege of serving as a technical assistance resource center for the U.S. Administration on Aging (AoA) since CHA’s inception (1). In addition, CHA has been the leader of the Falls Free©Initiative (2), a national collaborative effort to reduce falls among older adults.

With more than 10,000 baby boomers turning 65 each day (3), leveraging precious resources and cultivating innovative partnerships is critical for making a population health impact. As a national resource center, CHA identifies, develops, and disseminates best practices and tools for use by program implementers. We have a rich history of successful collaboration with aging services organizations, and over the past few years have thoughtfully expanded our network to include health care organizations and other private sector partners.

Citing solid evidence, proponents of evidence-based health promotion programs have long asserted that these interventions have a positive impact on health and wellness (4, 5), and the passage of the Patient Protection and Affordable Care Act (6) (ACA) in 2010 bolstered efforts already underway to engage those providing and paying for health care. In addition to increasing the quality and affordability of health insurance and lowering the rate of uninsured individuals, a number of ACA initiatives focus on improving patient outcomes and satisfaction. Evidence-based programs, particularly those emphasizing self-management, are well-positioned to serve as the carrot bridging community-based organizations, which have a history of successful and efficient program delivery, with the newly incentivized health care sector. Further supporting the value of this collaboration is a recent national study of Stanford University’s Chronic Disease Self-Management Program (7), finding many significant improvements aligned with the Institute of Healthcare Improvement’s Triple Aims of better health, better care, and lower cost (8, 9).

Evidence-based programs are being implemented in nearly every state, with hundreds of thousands of participants benefiting. In light of dwindling federal resources to support these programs and recognizing that there is no single “golden ticket” to program sustainability, program implementers are looking instead to a variety of blended funding streams. The rapid changes in health care delivery under the ACA have afforded an opportunity to integrate evidence-based programs into developing health systems and initiatives such as Accountable Care Organizations, Managed Care Organizations, Community-Based Care Transitions, and Patient-Centered Medical Homes. As a resource center, we recognize how essential it is to develop the acumen and skills necessary to form meaningful and mutually beneficial relationships for program reimbursement, and are committed to working with our national network of partners to expand this knowledge base.

To this end, CHA formed the Community-Integrated Health Care Workgroup in early 2014 to assist our network in their efforts to obtain reimbursement to sustain implementation of evidence-based programs. Participants include members of the aging services network from area agencies on aging, senior resource centers, and other settings. The objectives for the group are to: (1) develop specific definitions and parameters of community-integrated health care; (2) promote best practices in community-based organization/health care integration occurring in the aging network; and (3) identify barriers to this integration and potential steps address them.

In addition to various activities that fall under the scope of our role as AoA’s national resource center on chronic disease self-management education (CDSME) programs, NCOA’s Self-Management Alliance (SMA) is also a key conduit as we work toward our goal of improving the health of millions of older adults. The SMA promotes strategic collaboration among government, business, and non-profit organizations to achieve the goal of making evidence-based self-management an integral part of health care. It fosters information sharing, consensus development, research and demonstrations, communications, and public policy in support of nationwide scaling of self-management and other evidence-based programs. The SMA is involved in a series of efforts to better understand and delineate the value proposition of CDSME implementation for health systems and to identify “building blocks” for integrated community health systems with the goal of sustainable reimbursement for CDSME programs.

We also recognize the value of taking a “two venue” approach to program implementation, with participants able to select in-person or online workshops. NCOA distributes the online suite of Stanford University’s self-management programs, known as Better Choices, Better Health^®^ (including variants specific to diabetes, arthritis, cancer survivors, and caregiving). The opportunity to enroll in a workshop in-person or online is a considerable value-add for organizations, allowing them to cast a wider net as they engage partners and participants alike.

At NCOA, we are committed to providing national technical assistance, informed leadership, and strategic resources to advance the implementation and sustainability of evidence-based health promotion programs. A number of challenges remain, and working with our large network of partners to identify feasible solutions is at the top of our agenda. To meet the demands of an aging population and ensure access to these proven programs, a robust workforce of program facilitators is necessary. Given the paramount challenge of health systems and societies globally to support positive behavior change in an effort to tackle the preventable causes of chronic illness, strategies to boost participant engagement require additional research and experimentation. Developing the capacity to offer programs on a consistent basis with broad geographic reach is critically important to the success of our partnership with health care organizations; a statewide system with the capability to deliver programs to their members within a reasonable period of time and within close proximity to where they live and work is expected. We are confident that within these challenges exist opportunities for further innovation, collaboration, and impact, and are excited about what lies ahead.

## Conflict of Interest Statement

The authors declare that the research was conducted in the absence of any commercial or financial relationships that could be construed as a potential conflict of interest.

This paper is included in the Research Topic, “Evidence-Based Programming for Older Adults.” This Research Topic received partial funding from multiple government and private organizations/agencies; however, the views, findings, and conclusions in these articles are those of the authors and do not necessarily represent the official position of these organizations/agencies. All papers published in the Research Topic received peer review from members of the Frontiers in Public Health (Public Health Education and Promotion section) panel of Review Editors. Because this Research Topic represents work closely associated with a nationwide evidence-based movement in the US, many of the authors and/or Review Editors may have worked together previously in some fashion. Review Editors were purposively selected based on their expertise with evaluation and/or evidence-based programming for older adults. Review Editors were independent of named authors on any given article published in this volume.

## References

[1] Center for Healthy Aging. (2014). Available from: http://www.ncoa.org/improve-health/center-for-healthy-aging/

[2] Falls Free©Initiative. (2014). Available from: http://www.ncoa.org/improve-health/center-for-healthy-aging/falls-prevention/falls-free-initiative.html

[3] Pew Research Center. Baby Boomers Retire. (2010). Available from: http://www.pewresearch.org/daily-number/baby-boomers-retire/

[4] Beattie BL Whitelaw N Mettler M Turner D. A vision for older adults and health promotion. Am J Health Promot (2003) 18(2):200–4. 10.4278/0890-1171-18.2.200 14621420

[5] Bryant LL Altpeter A Whitelaw NA Evaluation of health promotion programs for older adults: an introduction. J Appl Gerontol (2006) 25(3):197–213 10.1177/0733464806288562

[6] Patient Protection and Affordable Care Act, 42 U.S.C. §18001. (2010). Available from: http://guides.nyu.edu/apaciting

[7] Ory MG Smith ML Ahn S Jiang L Kulinski KP Jiang N National Study of the Chronic Disease Self-Management Program: A Brief Overview. Washington, DC: National Council on Aging (2013). 10.1097/MLR.0b013e3182a95dd1 24113813

[8] Ory MG Ahn S Jiang L Smith ML Ritter PL Whitelaw N Success of a National Study of the Chronic Disease Self-Management Program: meeting the triple aim of health Care Reform. Med Care (2013) 51(11):992–8. 10.1097/MLR.0b013e3182a95dd1 24113813

[9] Berwick DM Nolan TW Whittington J. The triple aim: care, health, and cost. Health Aff (2008) 27:759–69. 10.1377/hlthaff.27.3.759 18474969

